# Bis[*N*,*N*′-bis­(2,6-diisopropyl­phen­yl)ethane-1,2-diimine]-1κ^2^ *N*,*N*′;2κ^2^ *N*,*N*′-tri-μ-trichlorido-1:2κ^6^ *Cl*:*Cl*-chlorido-1κ*Cl*-tetra­hydro­furan-2κ*O*-dichromium(II) dichloro­methane 4.5-solvate

**DOI:** 10.1107/S1600536809047266

**Published:** 2009-11-14

**Authors:** Stephan Peitz, Normen Peulecke, Bernd H. Müller, Anke Spannenberg, Uwe Rosenthal

**Affiliations:** a Leibniz-Institut für Katalyse e. V. an der Universität Rostock, Albert-Einstein-Strasse 29a, 18059 Rostock, Germany

## Abstract

In the mol­ecular structure of the title compound, [Cr_2_Cl_4_(C_26_H_36_N_2_)_2_(C_4_H_8_O)]·4.5CH_2_Cl_2_, the two Cr^II^ centers are bridged by three Cl atoms, forming a dinuclear complex. Each Cr^II^ center is coordinated by one chelating bis­(2,6-diisopropyl­phen­yl)ethane-1,2-diimine ligand *via* both N atoms. An additional chloride ion binds to one chromium center, whereas an additional tetra­hydro­furan mol­ecule coordinates to the second Cr^II^ center. The coordination geometry at each Cr^II^ center can be best described as distorted octa­hedral.

## Related literature

For a different crystal structure of the title compound from diethyl­ether, see: Kreisel *et al.* (2008 ▶). For binuclear chromium complexes, see: Dietel *et al.* (2006 ▶); Kreisel *et al.* (2007 ▶); Wagner *et al.* (2009 ▶). For a different product of the reaction described here, obtained by crystallization of the raw material from acetonitrile, see: Peitz *et al.* (2009 ▶).
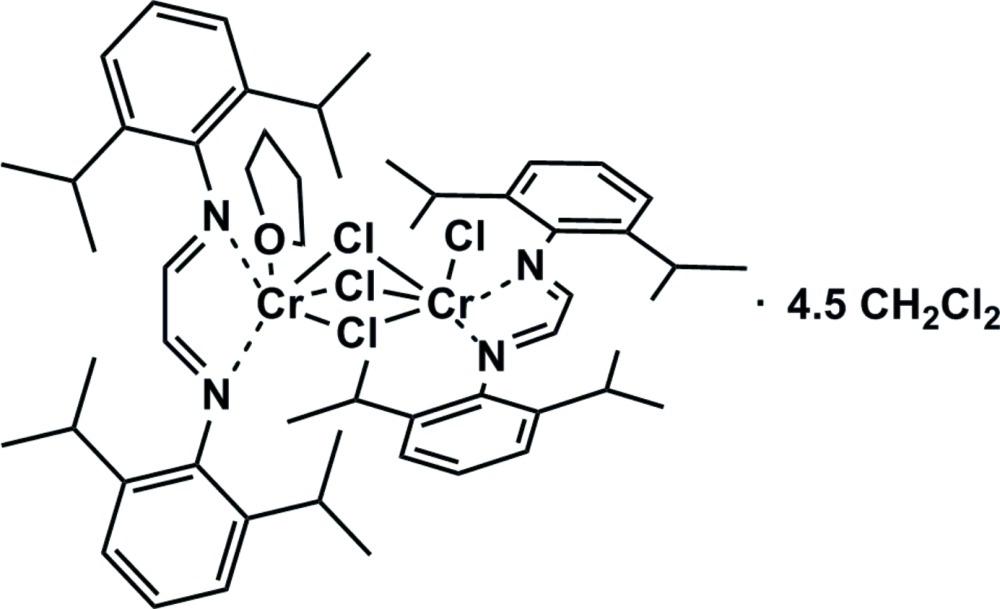


## Experimental

### 

#### Crystal data

[Cr_2_Cl_4_(C_26_H_36_N_2_)_2_(C_4_H_8_O)]·4.5CH_2_Cl_2_
*M* *_r_* = 1453.21
Monoclinic, 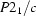

*a* = 18.2545 (6) Å
*b* = 15.4111 (4) Å
*c* = 27.3121 (7) Å
β = 105.160 (2)°
*V* = 7416.1 (4) Å^3^
*Z* = 4
Mo *K*α radiation
μ = 0.80 mm^−1^
*T* = 200 K
0.50 × 0.50 × 0.37 mm

#### Data collection

STOE IPDS II diffractometer
Absorption correction: numerical (**X-SHAPE** and **X-RED32**; Stoe & Cie, 2005 ▶) *T*_min_ = 0.672, *T*_max_ = 0.810
94968 measured reflections
13785 independent reflections
9450 reflections with *I* > 2σ(*I*)
*R*_int_ = 0.053

#### Refinement

*R*[*F* ^2^ > 2σ(*F* ^2^)] = 0.044
*wR*(*F* ^2^) = 0.123
*S* = 0.93
13785 reflections
747 parameters
20 restraints
H-atom parameters constrained
Δρ_max_ = 0.84 e Å^−3^
Δρ_min_ = −0.57 e Å^−3^

### 

Data collection: *X-AREA* (Stoe & Cie, 2005 ▶); cell refinement: *X-AREA*; data reduction: *X-AREA*; program(s) used to solve structure: *SHELXS97* (Sheldrick, 2008 ▶); program(s) used to refine structure: *SHELXL97* (Sheldrick, 2008 ▶); molecular graphics: *XP* in *SHELXTL* (Sheldrick, 2008 ▶); software used to prepare material for publication: *SHELXTL*.

## Supplementary Material

Crystal structure: contains datablocks I, global. DOI: 10.1107/S1600536809047266/im2155sup1.cif

Structure factors: contains datablocks I. DOI: 10.1107/S1600536809047266/im2155Isup2.hkl

Additional supplementary materials:  crystallographic information; 3D view; checkCIF report

## Figures and Tables

**Fig. 1.**
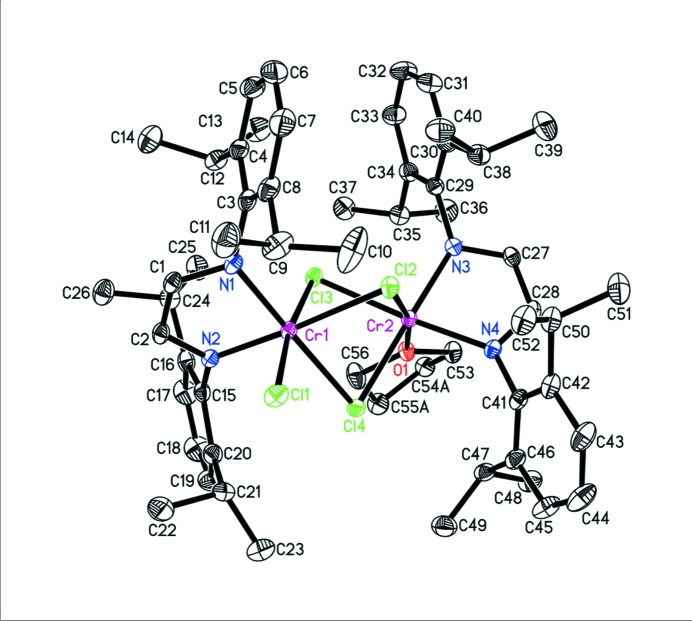
Molecular structure of the title compound showing the atom-labelling scheme. Thermal ellipsoids are drawn at the 30% probability level. Hydrogen atoms are omitted for clarity. For the disordered THF molecule atoms with higher occupancy are shown. The asymmetric unit additionally contains solvent molecules which are not depicted.

## supplementary crystallographic information

### Comment

Binuclear chromium complexes have been investigated concerning their structural and electronic features (Dietel *et al.*, 2006). Additionally, chromium-chromium interactions are of great interest due to the features of metal-metal multiple bonds. (Wagner *et al.*, 2009). With the same ligand used here, a binuclear chromium complex with the shortest metal-metal bond at that time was isolated (Kreisel *et al.*, 2007).

We became interested in chromium complexes with this type of ligand during our studies on binuclear chromium complexes. Therefore, we wanted to examine the *N,N'*-chelating diimine ligand in combination with chromium in order to find differences and similarities in coordination chemistry compared to other ligands forming binuclear complexes. We deployed a simple preparation procedure that is described here, to obtain the complex for structural investigations. Depending on the solvent used for crystallization of the raw material, mono- or binuclear complexes can be formed out of the educts (Peitz *et al.*, 2009).

The molecular structure of the title compound shows two chromium(II) centers that are bridged by three chlorine atoms to form a binuclear complex (Fig. 1). Each chromium is coordinated by one chelating diimine ligand, (*i*-Pr)_2_C_6_H_3_–NC(H)–C(H)N–C_6_H_3_(*i*-Pr)_2_, *via* both N atoms of each ligand. An additional chloride ion binds to one chromium center, whereas an additional THF molecule coordinates to the second chromium center. The coordination geometry at each chromium center can be best described as distorted octahedral. Cr1 deviates from the N1,C1,C2,N2 plane by 0.45 Å, Cr2 from the N3,C27,C28,N4 plane by 0.28 Å. The NCCN backbones of the ligands are twisted in an angle of 62.1 (1)° against each other. The asymmetric unit additionally contains 4.5 solvent molecules dichloromethane per dinuclear complex unit. The title compound was previously investigated by X-ray analysis but single crystals were obtained from a different solvent and besides two molecules [(C_26_H_36_N_2_)CrCl(µ-Cl)_3_Cr(THF)(C_26_H_36_N_2_)] they contained disordered diethyl ether molecules (space group *P*2_1_/*n*; a = 15.3679 (15), b = 40.969 (4), c = 22.124 (2) Å, β = 108.121 (2)°).

### Experimental

CrCl_2_(THF)_2_ (1.50 g, 5.66 mmol) and *N,N'*-bis(2,6- diisopropylphenyl)ethane-1,2-diimine (2.13 g, 5.66 mmol) were dissolved in 20 ml of dichloromethane at room temperature and stirred over night. After removal of all volatiles in vacuum the residue was washed with small amounts of *n*-hexane to yield 3.05 g (93%) of green complex. Crystallization in dichloromethane/*n*-hexane (1:1) yielded dark-green single crystals suitable for X-ray analysis.

### Refinement

All H atoms were placed in idealized positions with d(C—H) = 0.99 (CH_2_), 0.98 (CH_3_) and 0.95–1.00 Å (CH) and refined using a riding model with *U*_iso_(H) fixed at 1.5 *U*_eq_(C) for CH_3_ and 1.2 *U*_eq_(C) for CH_2_ and CH. All non-hydrogen atoms are refined anisotropically, except not fully occupied non-hydrogen atoms of the coordinated THF molecule which are refined isotropically with occupancies 0.64:0.36. Distance restraints (SADI in *SHELXL*) were used to improve the geometry of the disorded THF and the disordered CH_2_Cl_2_ molecule. It was also used to equalize two bonds within each of two aryl rings.

### Crystal data

**Table:**

[Cr_2_Cl_4_(C_26_H_36_N_2_)_2_(C_4_H_8_O)]·4.5CH_2_Cl_2_	*F*(000) = 3028
*M**_r_* = 1453.21	*D*_x_ = 1.302 Mg m^−^^3^
Monoclinic, *P*2_1_/*c*	Mo *K*α radiation, λ = 0.71073 Å
Hall symbol: -P 2ybc	Cell parameters from 13579 reflections
*a* = 18.2545 (6) Å	θ = 1.5–27.1°
*b* = 15.4111 (4) Å	µ = 0.80 mm^−^^1^
*c* = 27.3121 (7) Å	*T* = 200 K
β = 105.160 (2)°	Prism, dark-green
*V* = 7416.1 (4) Å^3^	0.50 × 0.50 × 0.37 mm
*Z* = 4	

### Data collection

**Table:**

STOE IPDS II diffractometer	13785 independent reflections
Radiation source: fine-focus sealed tube	9450 reflections with *I* > 2σ(*I*)
graphite	*R*_int_ = 0.053
ω scans	θ_max_ = 25.5°, θ_min_ = 1.5°
Absorption correction: numerical (*X-SHAPE* and *X-RED32*; Stoe & Cie, 2005)	*h* = −22→22
*T*_min_ = 0.672, *T*_max_ = 0.810	*k* = −18→18
94968 measured reflections	*l* = −33→31

### Refinement

**Table:**

Refinement on *F*^2^	Primary atom site location: structure-invariant direct methods
Least-squares matrix: full	Secondary atom site location: difference Fourier map
*R*[*F*^2^ > 2σ(*F*^2^)] = 0.044	Hydrogen site location: inferred from neighbouring sites
*wR*(*F*^2^) = 0.123	H-atom parameters constrained
*S* = 0.93	*w* = 1/[σ^2^(*F*_o_^2^) + (0.078*P*)^2^] where *P* = (*F*_o_^2^ + 2*F*_c_^2^)/3
13785 reflections	(Δ/σ)_max_ = 0.001
747 parameters	Δρ_max_ = 0.84 e Å^−^^3^
20 restraints	Δρ_min_ = −0.57 e Å^−^^3^

### Special details

**Table:**

Geometry. All e.s.d.'s (except the e.s.d. in the dihedral angle between two l.s. planes) are estimated using the full covariance matrix. The cell e.s.d.'s are taken into account individually in the estimation of e.s.d.'s in distances, angles and torsion angles; correlations between e.s.d.'s in cell parameters are only used when they are defined by crystal symmetry. An approximate (isotropic) treatment of cell e.s.d.'s is used for estimating e.s.d.'s involving l.s. planes.
Refinement. Refinement of *F*^2^ against ALL reflections. The weighted *R*-factor *wR* and goodness of fit *S* are based on *F*^2^, conventional *R*-factors *R* are based on *F*, with *F* set to zero for negative *F*^2^. The threshold expression of *F*^2^ > σ(*F*^2^) is used only for calculating *R*-factors(gt) *etc*. and is not relevant to the choice of reflections for refinement. *R*-factors based on *F*^2^ are statistically about twice as large as those based on *F*, and *R*- factors based on ALL data will be even larger.

### Fractional atomic coordinates and isotropic or equivalent isotropic displacement parameters (Å^2^)

**Table:**

	*x*	*y*	*z*	*U*_iso_*/*U*_eq_	Occ. (<1)
C1	0.11225 (16)	0.80860 (18)	0.29570 (11)	0.0283 (6)	
H1A	0.0823	0.8593	0.2863	0.034*	
C2	0.10700 (16)	0.75849 (18)	0.33679 (12)	0.0287 (6)	
H2A	0.0728	0.7726	0.3565	0.034*	
C3	0.15377 (16)	0.81698 (18)	0.21974 (12)	0.0300 (7)	
C4	0.09725 (17)	0.78284 (19)	0.17878 (12)	0.0333 (7)	
C5	0.0907 (2)	0.8165 (2)	0.13071 (14)	0.0444 (8)	
H5A	0.0531	0.7938	0.1028	0.053*	
C6	0.13718 (19)	0.8819 (2)	0.12255 (14)	0.0517 (10)	
H6A	0.1318	0.9039	0.0893	0.062*	
C7	0.1914 (2)	0.9151 (2)	0.16265 (15)	0.0494 (9)	
H7A	0.2232	0.9606	0.1567	0.059*	
C8	0.20147 (18)	0.8845 (2)	0.21175 (13)	0.0376 (8)	
C9	0.26128 (19)	0.9252 (2)	0.25446 (16)	0.0487 (9)	
H9A	0.2617	0.8930	0.2863	0.058*	
C10	0.3407 (2)	0.9179 (3)	0.2458 (2)	0.0756 (16)	
H10A	0.3523	0.8567	0.2414	0.113*	
H10B	0.3783	0.9418	0.2751	0.113*	
H10C	0.3423	0.9504	0.2153	0.113*	
C11	0.2431 (2)	1.0204 (3)	0.26245 (18)	0.0624 (11)	
H11A	0.1929	1.0244	0.2689	0.094*	
H11B	0.2433	1.0539	0.2320	0.094*	
H11C	0.2814	1.0439	0.2915	0.094*	
C12	0.04300 (17)	0.71206 (19)	0.18585 (13)	0.0359 (7)	
H12A	0.0628	0.6881	0.2208	0.043*	
C13	0.0397 (2)	0.6368 (2)	0.14810 (15)	0.0544 (10)	
H13A	0.0911	0.6147	0.1513	0.082*	
H13B	0.0182	0.6578	0.1134	0.082*	
H13C	0.0078	0.5902	0.1556	0.082*	
C14	−0.03609 (19)	0.7484 (2)	0.18194 (16)	0.0532 (10)	
H14A	−0.0325	0.7963	0.2061	0.080*	
H14B	−0.0684	0.7026	0.1898	0.080*	
H14C	−0.0581	0.7697	0.1474	0.080*	
C15	0.13473 (16)	0.61857 (18)	0.37644 (12)	0.0291 (6)	
C16	0.07189 (17)	0.56534 (19)	0.35457 (12)	0.0329 (7)	
C17	0.0601 (2)	0.4920 (2)	0.38173 (15)	0.0482 (9)	
H17A	0.0186	0.4546	0.3676	0.058*	
C18	0.1076 (3)	0.4733 (3)	0.42855 (16)	0.0586 (11)	
H18A	0.0995	0.4222	0.4459	0.070*	
C19	0.1666 (2)	0.5273 (2)	0.45041 (14)	0.0506 (9)	
H19A	0.1980	0.5139	0.4831	0.061*	
C20	0.18147 (18)	0.6019 (2)	0.42554 (12)	0.0362 (7)	
C21	0.24305 (19)	0.6649 (2)	0.45129 (12)	0.0403 (8)	
H21A	0.2649	0.6904	0.4245	0.048*	
C22	0.2082 (2)	0.7392 (3)	0.47484 (15)	0.0583 (11)	
H22A	0.1667	0.7655	0.4489	0.087*	
H22B	0.2471	0.7832	0.4882	0.087*	
H22C	0.1886	0.7166	0.5025	0.087*	
C23	0.3083 (2)	0.6230 (3)	0.49129 (15)	0.0630 (11)	
H23A	0.3303	0.5758	0.4757	0.095*	
H23B	0.2889	0.5997	0.5189	0.095*	
H23C	0.3473	0.6667	0.5049	0.095*	
C24	0.01653 (17)	0.5850 (2)	0.30430 (13)	0.0351 (7)	
H24A	0.0403	0.6285	0.2861	0.042*	
C25	−0.0048 (2)	0.5050 (2)	0.27030 (15)	0.0464 (9)	
H25A	0.0413	0.4790	0.2646	0.070*	
H25B	−0.0389	0.5222	0.2377	0.070*	
H25C	−0.0304	0.4626	0.2869	0.070*	
C26	−0.0562 (2)	0.6249 (2)	0.31290 (16)	0.0497 (9)	
H26A	−0.0433	0.6765	0.3343	0.075*	
H26B	−0.0814	0.5823	0.3297	0.075*	
H26C	−0.0905	0.6412	0.2802	0.075*	
C27	0.37043 (16)	0.46030 (18)	0.20108 (11)	0.0283 (6)	
H27A	0.3743	0.4304	0.1714	0.034*	
C28	0.43204 (16)	0.46895 (18)	0.24300 (11)	0.0285 (6)	
H28A	0.4803	0.4457	0.2433	0.034*	
C29	0.24730 (16)	0.50720 (19)	0.15721 (11)	0.0283 (6)	
C30	0.25609 (18)	0.5725 (2)	0.12378 (12)	0.0344 (7)	
C31	0.2010 (2)	0.5803 (2)	0.07764 (13)	0.0460 (9)	
H31A	0.2062	0.6245	0.0545	0.055*	
C32	0.1394 (2)	0.5258 (2)	0.06487 (13)	0.0516 (9)	
H32A	0.1025	0.5325	0.0333	0.062*	
C33	0.13153 (19)	0.4614 (2)	0.09806 (13)	0.0430 (8)	
H33A	0.0892	0.4233	0.0889	0.052*	
C34	0.18444 (17)	0.45078 (19)	0.14485 (12)	0.0329 (7)	
C35	0.17501 (18)	0.3773 (2)	0.17941 (13)	0.0355 (7)	
H35A	0.2073	0.3905	0.2142	0.043*	
C36	0.2038 (2)	0.2920 (2)	0.16180 (15)	0.0502 (9)	
H36A	0.2564	0.2994	0.1602	0.075*	
H36B	0.1720	0.2767	0.1281	0.075*	
H36C	0.2012	0.2457	0.1859	0.075*	
C37	0.09336 (19)	0.3675 (2)	0.18277 (15)	0.0458 (9)	
H37A	0.0761	0.4223	0.1942	0.069*	
H37B	0.0908	0.3214	0.2070	0.069*	
H37C	0.0607	0.3524	0.1493	0.069*	
C38	0.32372 (18)	0.6347 (2)	0.13481 (13)	0.0368 (7)	
H38A	0.3536	0.6250	0.1707	0.044*	
C39	0.3758 (2)	0.6154 (3)	0.10037 (16)	0.0577 (10)	
H39A	0.4188	0.6559	0.1082	0.086*	
H39B	0.3473	0.6223	0.0648	0.086*	
H39C	0.3948	0.5558	0.1061	0.086*	
C40	0.2986 (2)	0.7296 (2)	0.12971 (14)	0.0490 (9)	
H40A	0.2656	0.7410	0.1521	0.073*	
H40B	0.2706	0.7414	0.0945	0.073*	
H40C	0.3433	0.7673	0.1392	0.073*	
C41	0.48895 (16)	0.54084 (18)	0.31973 (12)	0.0305 (7)	
C42	0.53291 (16)	0.6055 (2)	0.30411 (13)	0.0355 (7)	
C43	0.60213 (18)	0.6276 (2)	0.33735 (16)	0.0490 (9)	
H43A	0.6333	0.6699	0.3274	0.059*	
C44	0.6262 (2)	0.5894 (3)	0.38420 (17)	0.0582 (11)	
H44A	0.6742	0.6045	0.4060	0.070*	
C45	0.5811 (2)	0.5293 (2)	0.39982 (15)	0.0509 (10)	
H45A	0.5977	0.5052	0.4329	0.061*	
C46	0.51160 (17)	0.5033 (2)	0.36812 (12)	0.0352 (7)	
C47	0.46645 (18)	0.4333 (2)	0.38578 (12)	0.0344 (7)	
H47A	0.4147	0.4314	0.3617	0.041*	
C48	0.5038 (2)	0.3447 (2)	0.38485 (14)	0.0466 (9)	
H48A	0.5099	0.3336	0.3508	0.070*	
H48B	0.4717	0.2995	0.3937	0.070*	
H48C	0.5537	0.3442	0.4095	0.070*	
C49	0.4573 (2)	0.4510 (3)	0.43872 (13)	0.0520 (9)	
H49A	0.4334	0.5078	0.4392	0.078*	
H49B	0.5073	0.4507	0.4632	0.078*	
H49C	0.4254	0.4059	0.4478	0.078*	
C50	0.50759 (18)	0.65330 (19)	0.25420 (13)	0.0358 (7)	
H50A	0.4547	0.6341	0.2372	0.043*	
C51	0.5571 (2)	0.6329 (2)	0.21852 (17)	0.0537 (10)	
H51A	0.5579	0.5701	0.2131	0.081*	
H51B	0.6089	0.6535	0.2336	0.081*	
H51C	0.5364	0.6620	0.1859	0.081*	
C52	0.5057 (2)	0.7517 (2)	0.26284 (15)	0.0451 (9)	
H52A	0.4734	0.7642	0.2856	0.068*	
H52B	0.4851	0.7809	0.2303	0.068*	
H52C	0.5572	0.7726	0.2782	0.068*	
C53	0.32147 (17)	0.33239 (18)	0.28926 (13)	0.0347 (7)	
H53A	0.3755	0.3283	0.3084	0.042*	0.637 (11)
H53B	0.3178	0.3259	0.2526	0.042*	0.637 (11)
H53C	0.3772	0.3372	0.2950	0.042*	0.363 (11)
H53D	0.2990	0.3133	0.2539	0.042*	0.363 (11)
C54A	0.2745 (3)	0.2634 (3)	0.3068 (2)	0.0399 (16)*	0.637 (11)
H54A	0.3048	0.2104	0.3182	0.048*	0.637 (11)
H54B	0.2290	0.2480	0.2795	0.048*	0.637 (11)
C55A	0.2527 (4)	0.3075 (2)	0.3508 (2)	0.0470 (18)*	0.637 (11)
H55A	0.2075	0.2801	0.3578	0.056*	0.637 (11)
H55B	0.2951	0.3063	0.3820	0.056*	0.637 (11)
C54B	0.3016 (5)	0.2697 (5)	0.3268 (4)	0.038 (3)*	0.363 (11)
H54C	0.2989	0.2093	0.3141	0.045*	0.363 (11)
H54D	0.3391	0.2728	0.3603	0.045*	0.363 (11)
C55B	0.2242 (5)	0.3018 (2)	0.3298 (5)	0.041 (3)*	0.363 (11)
H55C	0.1837	0.2837	0.2998	0.049*	0.363 (11)
H55D	0.2118	0.2808	0.3610	0.049*	0.363 (11)
C56	0.2358 (2)	0.3993 (2)	0.33100 (16)	0.0473 (9)	
H56A	0.1825	0.4045	0.3106	0.057*	0.637 (11)
H56B	0.2446	0.4413	0.3594	0.057*	0.637 (11)
H56C	0.1871	0.4297	0.3169	0.057*	0.363 (11)
H56D	0.2578	0.4197	0.3662	0.057*	0.363 (11)
N1	0.16121 (12)	0.78254 (14)	0.26992 (9)	0.0253 (5)	
N2	0.15262 (12)	0.68918 (14)	0.34728 (9)	0.0244 (5)	
N3	0.30513 (13)	0.49636 (14)	0.20455 (9)	0.0245 (5)	
N4	0.42064 (12)	0.51140 (14)	0.28312 (9)	0.0252 (5)	
O1	0.28868 (11)	0.41535 (12)	0.29936 (8)	0.0286 (4)	
Cl1	0.29709 (4)	0.80183 (5)	0.36299 (3)	0.03575 (18)	
Cl2	0.31971 (4)	0.68646 (4)	0.25774 (3)	0.02920 (16)	
Cl3	0.18000 (4)	0.57077 (4)	0.25873 (3)	0.02682 (16)	
Cl4	0.32064 (4)	0.58807 (4)	0.35821 (3)	0.02876 (16)	
Cr1	0.23158 (2)	0.69493 (3)	0.309904 (17)	0.02299 (11)	
Cr2	0.31100 (2)	0.53728 (3)	0.274554 (17)	0.02229 (11)	
C58	0.1798 (3)	0.9680 (3)	0.4190 (2)	0.0871 (16)	
H58A	0.2090	0.9778	0.4546	0.105*	
H58B	0.2015	0.9166	0.4062	0.105*	
Cl7	0.18933 (10)	1.05833 (14)	0.38280 (8)	0.1275 (7)	
Cl8	0.08513 (7)	0.94725 (8)	0.41733 (5)	0.0792 (4)	
C59	0.4061 (4)	−0.0072 (4)	0.4289 (3)	0.110 (2)	
H59A	0.3688	−0.0406	0.4028	0.132*	
H59B	0.4565	−0.0346	0.4334	0.132*	
Cl9	0.40933 (11)	0.09914 (15)	0.40844 (8)	0.1363 (7)	
Cl10	0.38101 (10)	−0.01068 (14)	0.48510 (6)	0.1197 (6)	
C60	0.5119 (3)	0.2487 (3)	0.09401 (19)	0.0709 (13)	
H60A	0.5653	0.2533	0.1144	0.085*	
H60B	0.4897	0.1960	0.1051	0.085*	
Cl11	0.46194 (10)	0.33895 (10)	0.10523 (6)	0.0983 (5)	
Cl12	0.50985 (11)	0.23883 (15)	0.03107 (7)	0.1319 (7)	
C61	0.2913 (4)	0.2102 (5)	0.4963 (3)	0.087 (2)	0.75
H61A	0.3457	0.2110	0.5149	0.105*	0.75
H61B	0.2874	0.1793	0.4639	0.105*	0.75
Cl13	0.26315 (18)	0.31482 (15)	0.48220 (9)	0.1267 (9)	0.75
Cl14	0.24382 (15)	0.1508 (2)	0.53092 (11)	0.1330 (9)	0.75
C57	0.0102 (3)	0.1422 (3)	0.0331 (3)	0.109 (3)	0.75
H57A	−0.0094	0.1213	−0.0022	0.130*	0.38
H57B	−0.0232	0.1190	0.0534	0.130*	0.38
H57C	−0.0165	0.1045	0.0047	0.130*	0.37
H57D	−0.0175	0.1388	0.0598	0.130*	0.37
Cl5	0.10128 (19)	0.10275 (16)	0.05752 (8)	0.1279 (9)	0.75
Cl6A	0.0066 (4)	0.2544 (2)	0.0336 (2)	0.0962 (17)	0.38
Cl6B	0.0067 (6)	0.2481 (3)	0.01156 (19)	0.114 (3)	0.37

### Atomic displacement parameters (Å^2^)

**Table:**

	*U*^11^	*U*^22^	*U*^33^	*U*^12^	*U*^13^	*U*^23^
C1	0.0267 (14)	0.0192 (13)	0.0375 (17)	0.0036 (11)	0.0057 (12)	−0.0012 (12)
C2	0.0257 (14)	0.0226 (14)	0.0375 (17)	0.0015 (11)	0.0079 (13)	−0.0053 (13)
C3	0.0302 (15)	0.0225 (14)	0.0378 (17)	0.0078 (12)	0.0097 (13)	0.0032 (12)
C4	0.0345 (16)	0.0287 (15)	0.0356 (17)	0.0083 (13)	0.0070 (14)	0.0018 (13)
C5	0.051 (2)	0.0391 (19)	0.0396 (19)	0.0096 (16)	0.0060 (16)	0.0052 (15)
C6	0.063 (2)	0.051 (2)	0.044 (2)	0.0118 (19)	0.0200 (19)	0.0166 (18)
C7	0.049 (2)	0.0405 (19)	0.063 (3)	0.0049 (16)	0.0222 (19)	0.0194 (18)
C8	0.0329 (16)	0.0277 (16)	0.052 (2)	0.0050 (13)	0.0106 (15)	0.0115 (15)
C9	0.0369 (18)	0.0398 (19)	0.064 (2)	−0.0119 (15)	0.0041 (17)	0.0189 (18)
C10	0.035 (2)	0.033 (2)	0.153 (5)	−0.0033 (16)	0.015 (2)	−0.003 (3)
C11	0.045 (2)	0.060 (3)	0.080 (3)	−0.0005 (18)	0.012 (2)	−0.013 (2)
C12	0.0362 (16)	0.0291 (16)	0.0370 (18)	0.0000 (13)	0.0003 (14)	−0.0013 (13)
C13	0.063 (2)	0.040 (2)	0.052 (2)	−0.0024 (18)	0.0002 (19)	−0.0084 (17)
C14	0.0385 (19)	0.048 (2)	0.070 (3)	0.0004 (16)	0.0084 (18)	0.0021 (19)
C15	0.0330 (15)	0.0246 (14)	0.0330 (16)	0.0058 (12)	0.0145 (13)	0.0014 (12)
C16	0.0364 (16)	0.0251 (15)	0.0417 (18)	0.0003 (12)	0.0180 (14)	−0.0017 (13)
C17	0.059 (2)	0.0343 (18)	0.057 (2)	−0.0086 (16)	0.0258 (19)	0.0038 (17)
C18	0.081 (3)	0.042 (2)	0.059 (3)	−0.001 (2)	0.028 (2)	0.0199 (19)
C19	0.064 (2)	0.049 (2)	0.040 (2)	0.0071 (19)	0.0140 (18)	0.0148 (17)
C20	0.0411 (17)	0.0339 (17)	0.0353 (18)	0.0079 (13)	0.0128 (14)	0.0031 (14)
C21	0.0426 (18)	0.0478 (19)	0.0281 (17)	0.0051 (15)	0.0049 (14)	−0.0005 (15)
C22	0.061 (2)	0.061 (3)	0.048 (2)	0.007 (2)	0.0053 (19)	−0.016 (2)
C23	0.053 (2)	0.082 (3)	0.046 (2)	0.011 (2)	−0.0017 (19)	0.007 (2)
C24	0.0346 (16)	0.0270 (15)	0.0449 (19)	−0.0076 (13)	0.0124 (14)	−0.0034 (14)
C25	0.0438 (19)	0.0405 (19)	0.056 (2)	−0.0106 (15)	0.0160 (17)	−0.0133 (17)
C26	0.0396 (19)	0.043 (2)	0.064 (2)	0.0000 (15)	0.0099 (18)	−0.0086 (18)
C27	0.0343 (15)	0.0219 (14)	0.0321 (16)	0.0007 (12)	0.0148 (13)	−0.0023 (12)
C28	0.0268 (14)	0.0226 (14)	0.0387 (17)	0.0043 (11)	0.0135 (13)	0.0016 (13)
C29	0.0310 (15)	0.0283 (15)	0.0254 (15)	0.0022 (12)	0.0068 (12)	−0.0032 (12)
C30	0.0400 (17)	0.0340 (16)	0.0309 (17)	0.0021 (13)	0.0123 (14)	0.0003 (13)
C31	0.054 (2)	0.049 (2)	0.0330 (18)	−0.0027 (17)	0.0092 (16)	0.0063 (16)
C32	0.052 (2)	0.063 (2)	0.0325 (19)	−0.0046 (18)	−0.0027 (16)	0.0019 (18)
C33	0.0412 (18)	0.0449 (19)	0.0382 (19)	−0.0081 (15)	0.0024 (15)	−0.0036 (16)
C34	0.0352 (16)	0.0320 (16)	0.0317 (17)	−0.0023 (13)	0.0091 (13)	−0.0064 (13)
C35	0.0378 (17)	0.0306 (16)	0.0373 (18)	−0.0057 (13)	0.0084 (14)	−0.0030 (14)
C36	0.058 (2)	0.0347 (19)	0.058 (2)	−0.0009 (16)	0.0138 (19)	−0.0044 (17)
C37	0.0424 (19)	0.044 (2)	0.052 (2)	−0.0117 (15)	0.0149 (17)	−0.0023 (17)
C38	0.0414 (17)	0.0353 (17)	0.0349 (17)	−0.0031 (14)	0.0122 (14)	0.0066 (14)
C39	0.063 (2)	0.058 (2)	0.062 (3)	−0.003 (2)	0.034 (2)	0.001 (2)
C40	0.061 (2)	0.0380 (19)	0.046 (2)	−0.0025 (17)	0.0093 (18)	0.0079 (16)
C41	0.0255 (14)	0.0257 (14)	0.0380 (17)	0.0048 (12)	0.0044 (13)	−0.0009 (13)
C42	0.0257 (15)	0.0305 (16)	0.050 (2)	0.0010 (12)	0.0099 (14)	0.0006 (14)
C43	0.0293 (17)	0.0399 (19)	0.073 (3)	−0.0046 (14)	0.0045 (17)	0.0051 (18)
C44	0.0334 (18)	0.054 (2)	0.073 (3)	−0.0071 (17)	−0.0111 (18)	−0.001 (2)
C45	0.0426 (19)	0.048 (2)	0.050 (2)	0.0053 (16)	−0.0097 (17)	0.0042 (18)
C46	0.0328 (16)	0.0322 (16)	0.0372 (18)	0.0083 (13)	0.0030 (14)	0.0011 (14)
C47	0.0375 (16)	0.0321 (16)	0.0300 (16)	0.0071 (13)	0.0024 (13)	0.0047 (13)
C48	0.052 (2)	0.0364 (18)	0.046 (2)	0.0121 (16)	0.0040 (17)	0.0030 (16)
C49	0.063 (2)	0.053 (2)	0.037 (2)	0.0117 (18)	0.0082 (18)	0.0021 (17)
C50	0.0317 (16)	0.0276 (16)	0.050 (2)	0.0001 (13)	0.0139 (14)	0.0021 (14)
C51	0.053 (2)	0.045 (2)	0.073 (3)	0.0095 (17)	0.034 (2)	0.012 (2)
C52	0.0410 (18)	0.0303 (17)	0.064 (2)	−0.0002 (14)	0.0132 (17)	0.0022 (16)
C53	0.0394 (17)	0.0208 (14)	0.0463 (19)	0.0051 (12)	0.0155 (15)	0.0010 (13)
C56	0.056 (2)	0.0370 (19)	0.062 (2)	0.0029 (16)	0.0383 (19)	0.0086 (17)
N1	0.0235 (11)	0.0173 (11)	0.0332 (13)	0.0006 (9)	0.0039 (10)	0.0007 (10)
N2	0.0236 (11)	0.0206 (11)	0.0277 (12)	−0.0004 (9)	0.0045 (10)	−0.0035 (10)
N3	0.0274 (12)	0.0202 (11)	0.0270 (13)	−0.0020 (9)	0.0089 (10)	−0.0008 (10)
N4	0.0242 (11)	0.0202 (11)	0.0310 (13)	0.0022 (9)	0.0069 (10)	0.0030 (10)
O1	0.0327 (10)	0.0211 (10)	0.0356 (11)	0.0031 (8)	0.0153 (9)	0.0027 (9)
Cl1	0.0334 (4)	0.0273 (4)	0.0410 (4)	−0.0039 (3)	−0.0003 (3)	−0.0055 (3)
Cl2	0.0301 (3)	0.0215 (3)	0.0385 (4)	0.0020 (3)	0.0135 (3)	0.0032 (3)
Cl3	0.0232 (3)	0.0227 (3)	0.0333 (4)	0.0014 (3)	0.0052 (3)	−0.0037 (3)
Cl4	0.0309 (3)	0.0269 (3)	0.0268 (4)	0.0074 (3)	0.0046 (3)	0.0000 (3)
Cr1	0.0216 (2)	0.0182 (2)	0.0281 (2)	0.00189 (17)	0.00455 (18)	−0.00073 (18)
Cr2	0.0223 (2)	0.0185 (2)	0.0265 (2)	0.00255 (17)	0.00712 (18)	0.00039 (18)
C58	0.063 (3)	0.075 (3)	0.111 (4)	0.007 (2)	0.001 (3)	0.001 (3)
Cl7	0.1034 (12)	0.1551 (17)	0.1255 (14)	−0.0021 (11)	0.0324 (11)	0.0440 (13)
Cl8	0.0656 (7)	0.0764 (8)	0.0899 (9)	0.0062 (6)	0.0102 (6)	−0.0126 (7)
C59	0.098 (4)	0.098 (4)	0.137 (6)	−0.005 (3)	0.034 (4)	−0.048 (4)
Cl9	0.1284 (14)	0.1613 (18)	0.1273 (15)	−0.0241 (13)	0.0480 (12)	0.0590 (13)
Cl10	0.1108 (12)	0.1592 (16)	0.0853 (10)	−0.0175 (11)	0.0189 (9)	0.0503 (11)
C60	0.055 (2)	0.060 (3)	0.095 (4)	−0.003 (2)	0.015 (2)	0.004 (3)
Cl11	0.1274 (12)	0.0849 (9)	0.0944 (10)	0.0328 (8)	0.0499 (9)	0.0142 (8)
Cl12	0.1228 (13)	0.1706 (18)	0.1001 (12)	0.0383 (13)	0.0251 (10)	−0.0424 (12)
C61	0.078 (4)	0.091 (5)	0.099 (5)	0.002 (4)	0.035 (4)	0.013 (4)
Cl13	0.186 (3)	0.0968 (15)	0.0962 (15)	0.0382 (16)	0.0347 (16)	0.0019 (12)
Cl14	0.134 (2)	0.156 (2)	0.140 (2)	−0.0201 (17)	0.0911 (18)	0.0088 (18)
C57	0.109 (6)	0.147 (8)	0.081 (5)	−0.062 (6)	0.045 (5)	0.006 (5)
Cl5	0.218 (3)	0.1073 (16)	0.0804 (13)	0.0387 (17)	0.0786 (17)	0.0059 (12)
Cl6A	0.085 (3)	0.091 (3)	0.104 (5)	−0.013 (2)	0.009 (4)	−0.027 (2)
Cl6B	0.145 (5)	0.133 (5)	0.061 (3)	−0.024 (4)	0.020 (4)	−0.017 (3)

### Geometric parameters (Å, °)

**Table:**

C1—N1	1.336 (4)	C39—H39B	0.9800
C1—C2	1.386 (4)	C39—H39C	0.9800
C1—H1A	0.9500	C40—H40A	0.9800
C2—N2	1.339 (3)	C40—H40B	0.9800
C2—H2A	0.9500	C40—H40C	0.9800
C3—C8	1.410 (4)	C41—C46	1.402 (4)
C3—C4	1.410 (4)	C41—C42	1.413 (4)
C3—N1	1.443 (4)	C41—N4	1.452 (4)
C4—C5	1.388 (5)	C42—C43	1.392 (4)
C4—C12	1.519 (4)	C42—C50	1.512 (5)
C5—C6	1.372 (4)	C43—C44	1.372 (6)
C5—H5A	0.9500	C43—H43A	0.9500
C6—C7	1.369 (4)	C44—C45	1.379 (6)
C6—H6A	0.9500	C44—H44A	0.9500
C7—C8	1.388 (5)	C45—C46	1.395 (4)
C7—H7A	0.9500	C45—H45A	0.9500
C8—C9	1.510 (5)	C46—C47	1.512 (5)
C9—C11	1.532 (5)	C47—C49	1.523 (5)
C9—C10	1.534 (5)	C47—C48	1.530 (4)
C9—H9A	1.0000	C47—H47A	1.0000
C10—H10A	0.9800	C48—H48A	0.9800
C10—H10B	0.9800	C48—H48B	0.9800
C10—H10C	0.9800	C48—H48C	0.9800
C11—H11A	0.9800	C49—H49A	0.9800
C11—H11B	0.9800	C49—H49B	0.9800
C11—H11C	0.9800	C49—H49C	0.9800
C12—C14	1.526 (5)	C50—C51	1.526 (5)
C12—C13	1.542 (5)	C50—C52	1.536 (4)
C12—H12A	1.0000	C50—H50A	1.0000
C13—H13A	0.9800	C51—H51A	0.9800
C13—H13B	0.9800	C51—H51B	0.9800
C13—H13C	0.9800	C51—H51C	0.9800
C14—H14A	0.9800	C52—H52A	0.9800
C14—H14B	0.9800	C52—H52B	0.9800
C14—H14C	0.9800	C52—H52C	0.9800
C15—C16	1.410 (4)	C53—O1	1.468 (3)
C15—C20	1.412 (4)	C53—C54A	1.519 (3)
C15—N2	1.436 (4)	C53—C54B	1.520 (3)
C16—C17	1.399 (5)	C53—H53A	0.9900
C16—C24	1.508 (4)	C53—H53B	0.9900
C17—C18	1.375 (6)	C53—H53C	0.9900
C17—H17A	0.9500	C53—H53D	0.9900
C18—C19	1.368 (6)	C54A—C55A	1.520 (3)
C18—H18A	0.9500	C54A—H54A	0.9900
C19—C20	1.397 (5)	C54A—H54B	0.9900
C19—H19A	0.9500	C55A—C56	1.518 (3)
C20—C21	1.513 (5)	C55A—H55A	0.9900
C21—C22	1.531 (5)	C55A—H55B	0.9900
C21—C23	1.532 (5)	C54B—C55B	1.520 (3)
C21—H21A	1.0000	C54B—H54C	0.9900
C22—H22A	0.9800	C54B—H54D	0.9900
C22—H22B	0.9800	C55B—C56	1.518 (3)
C22—H22C	0.9800	C55B—H55C	0.9900
C23—H23A	0.9800	C55B—H55D	0.9900
C23—H23B	0.9800	C56—O1	1.475 (4)
C23—H23C	0.9800	C56—H56A	0.9900
C24—C25	1.531 (4)	C56—H56B	0.9900
C24—C26	1.536 (5)	C56—H56C	0.9900
C24—H24A	1.0000	C56—H56D	0.9900
C25—H25A	0.9800	N1—Cr1	1.983 (2)
C25—H25B	0.9800	N2—Cr1	1.974 (2)
C25—H25C	0.9800	N3—Cr2	1.989 (2)
C26—H26A	0.9800	N4—Cr2	1.994 (2)
C26—H26B	0.9800	O1—Cr2	2.0737 (19)
C26—H26C	0.9800	Cl1—Cr1	2.3107 (8)
C27—N3	1.341 (4)	Cl2—Cr2	2.3579 (8)
C27—C28	1.386 (4)	Cl2—Cr1	2.4153 (9)
C27—H27A	0.9500	Cl3—Cr2	2.3730 (8)
C28—N4	1.338 (4)	Cl3—Cr1	2.4110 (8)
C28—H28A	0.9500	Cl4—Cr2	2.3778 (8)
C29—C30	1.395 (4)	Cl4—Cr1	2.4441 (8)
C29—C34	1.409 (4)	C58—Cl7	1.742 (6)
C29—N3	1.449 (4)	C58—Cl8	1.747 (5)
C30—C31	1.397 (5)	C58—H58A	0.9900
C30—C38	1.530 (4)	C58—H58B	0.9900
C31—C32	1.373 (4)	C59—Cl10	1.714 (7)
C31—H31A	0.9500	C59—Cl9	1.738 (7)
C32—C33	1.377 (4)	C59—H59A	0.9900
C32—H32A	0.9500	C59—H59B	0.9900
C33—C34	1.395 (4)	C60—Cl12	1.716 (5)
C33—H33A	0.9500	C60—Cl11	1.733 (5)
C34—C35	1.513 (4)	C60—H60A	0.9900
C35—C37	1.525 (5)	C60—H60B	0.9900
C35—C36	1.537 (5)	C61—Cl13	1.705 (7)
C35—H35A	1.0000	C61—Cl14	1.708 (7)
C36—H36A	0.9800	C61—H61A	0.9900
C36—H36B	0.9800	C61—H61B	0.9900
C36—H36C	0.9800	C57—Cl6A	1.730 (5)
C37—H37A	0.9800	C57—Cl6B	1.731 (5)
C37—H37B	0.9800	C57—Cl5	1.732 (4)
C37—H37C	0.9800	C57—H57A	0.9900
C38—C40	1.529 (5)	C57—H57B	0.9900
C38—C39	1.532 (5)	C57—H57C	0.9900
C38—H38A	1.0000	C57—H57D	0.9900
C39—H39A	0.9800		
			
N1—C1—C2	117.2 (2)	C45—C46—C41	117.6 (3)
N1—C1—H1A	121.4	C45—C46—C47	119.4 (3)
C2—C1—H1A	121.4	C41—C46—C47	123.0 (3)
N2—C2—C1	116.6 (3)	C46—C47—C49	112.3 (3)
N2—C2—H2A	121.7	C46—C47—C48	110.5 (3)
C1—C2—H2A	121.7	C49—C47—C48	109.6 (3)
C8—C3—C4	120.4 (3)	C46—C47—H47A	108.1
C8—C3—N1	120.7 (3)	C49—C47—H47A	108.1
C4—C3—N1	118.9 (3)	C48—C47—H47A	108.1
C5—C4—C3	118.4 (3)	C47—C48—H48A	109.5
C5—C4—C12	119.4 (3)	C47—C48—H48B	109.5
C3—C4—C12	122.2 (3)	H48A—C48—H48B	109.5
C6—C5—C4	121.6 (3)	C47—C48—H48C	109.5
C6—C5—H5A	119.2	H48A—C48—H48C	109.5
C4—C5—H5A	119.2	H48B—C48—H48C	109.5
C7—C6—C5	119.6 (3)	C47—C49—H49A	109.5
C7—C6—H6A	120.2	C47—C49—H49B	109.5
C5—C6—H6A	120.2	H49A—C49—H49B	109.5
C6—C7—C8	122.0 (3)	C47—C49—H49C	109.5
C6—C7—H7A	119.0	H49A—C49—H49C	109.5
C8—C7—H7A	119.0	H49B—C49—H49C	109.5
C7—C8—C3	118.0 (3)	C42—C50—C51	112.5 (3)
C7—C8—C9	119.4 (3)	C42—C50—C52	110.9 (3)
C3—C8—C9	122.6 (3)	C51—C50—C52	110.0 (3)
C8—C9—C11	111.6 (3)	C42—C50—H50A	107.7
C8—C9—C10	111.8 (4)	C51—C50—H50A	107.7
C11—C9—C10	110.0 (3)	C52—C50—H50A	107.7
C8—C9—H9A	107.7	C50—C51—H51A	109.5
C11—C9—H9A	107.7	C50—C51—H51B	109.5
C10—C9—H9A	107.7	H51A—C51—H51B	109.5
C9—C10—H10A	109.5	C50—C51—H51C	109.5
C9—C10—H10B	109.5	H51A—C51—H51C	109.5
H10A—C10—H10B	109.5	H51B—C51—H51C	109.5
C9—C10—H10C	109.5	C50—C52—H52A	109.5
H10A—C10—H10C	109.5	C50—C52—H52B	109.5
H10B—C10—H10C	109.5	H52A—C52—H52B	109.5
C9—C11—H11A	109.5	C50—C52—H52C	109.5
C9—C11—H11B	109.5	H52A—C52—H52C	109.5
H11A—C11—H11B	109.5	H52B—C52—H52C	109.5
C9—C11—H11C	109.5	O1—C53—C54A	105.0 (3)
H11A—C11—H11C	109.5	O1—C53—C54B	104.3 (4)
H11B—C11—H11C	109.5	O1—C53—H53A	110.8
C4—C12—C14	111.3 (3)	C54A—C53—H53A	110.8
C4—C12—C13	111.6 (3)	C54B—C53—H53A	89.0
C14—C12—C13	111.0 (3)	O1—C53—H53B	110.8
C4—C12—H12A	107.6	C54A—C53—H53B	110.8
C14—C12—H12A	107.6	C54B—C53—H53B	130.7
C13—C12—H12A	107.6	H53A—C53—H53B	108.8
C12—C13—H13A	109.5	O1—C53—H53C	110.9
C12—C13—H13B	109.5	C54A—C53—H53C	130.0
H13A—C13—H13B	109.5	C54B—C53—H53C	110.9
C12—C13—H13C	109.5	H53B—C53—H53C	87.9
H13A—C13—H13C	109.5	O1—C53—H53D	110.9
H13B—C13—H13C	109.5	C54A—C53—H53D	88.6
C12—C14—H14A	109.5	C54B—C53—H53D	110.9
C12—C14—H14B	109.5	H53A—C53—H53D	126.8
H14A—C14—H14B	109.5	H53C—C53—H53D	108.9
C12—C14—H14C	109.5	C53—C54A—C55A	102.9 (3)
H14A—C14—H14C	109.5	C53—C54A—H54A	111.2
H14B—C14—H14C	109.5	C55A—C54A—H54A	111.2
C16—C15—C20	121.3 (3)	C53—C54A—H54B	111.2
C16—C15—N2	118.9 (3)	C55A—C54A—H54B	111.2
C20—C15—N2	119.8 (3)	H54A—C54A—H54B	109.1
C17—C16—C15	117.8 (3)	C56—C55A—C54A	101.9 (4)
C17—C16—C24	119.1 (3)	C56—C55A—H55A	111.4
C15—C16—C24	123.1 (3)	C54A—C55A—H55A	111.4
C18—C17—C16	121.0 (3)	C56—C55A—H55B	111.4
C18—C17—H17A	119.5	C54A—C55A—H55B	111.4
C16—C17—H17A	119.5	H55A—C55A—H55B	109.3
C19—C18—C17	120.7 (3)	C55B—C54B—C53	102.7 (5)
C19—C18—H18A	119.6	C55B—C54B—H54C	111.2
C17—C18—H18A	119.6	C53—C54B—H54C	111.2
C18—C19—C20	121.3 (3)	C55B—C54B—H54D	111.2
C18—C19—H19A	119.4	C53—C54B—H54D	111.2
C20—C19—H19A	119.4	H54C—C54B—H54D	109.1
C19—C20—C15	117.7 (3)	C56—C55B—C54B	101.3 (5)
C19—C20—C21	121.2 (3)	C56—C55B—H55C	111.5
C15—C20—C21	121.0 (3)	C54B—C55B—H55C	111.5
C20—C21—C22	109.6 (3)	C56—C55B—H55D	111.5
C20—C21—C23	113.9 (3)	C54B—C55B—H55D	111.5
C22—C21—C23	110.3 (3)	H55C—C55B—H55D	109.3
C20—C21—H21A	107.6	O1—C56—C55A	105.2 (3)
C22—C21—H21A	107.6	O1—C56—C55B	105.2 (4)
C23—C21—H21A	107.6	O1—C56—H56A	110.7
C21—C22—H22A	109.5	C55A—C56—H56A	110.7
C21—C22—H22B	109.5	C55B—C56—H56A	87.5
H22A—C22—H22B	109.5	O1—C56—H56B	110.7
C21—C22—H22C	109.5	C55A—C56—H56B	110.7
H22A—C22—H22C	109.5	C55B—C56—H56B	131.1
H22B—C22—H22C	109.5	H56A—C56—H56B	108.8
C21—C23—H23A	109.5	O1—C56—H56C	110.7
C21—C23—H23B	109.5	C55A—C56—H56C	131.1
H23A—C23—H23B	109.5	C55B—C56—H56C	110.7
C21—C23—H23C	109.5	H56B—C56—H56C	86.5
H23A—C23—H23C	109.5	O1—C56—H56D	110.7
H23B—C23—H23C	109.5	C55A—C56—H56D	87.5
C16—C24—C25	113.2 (3)	C55B—C56—H56D	110.7
C16—C24—C26	110.0 (3)	H56A—C56—H56D	127.7
C25—C24—C26	108.8 (3)	H56C—C56—H56D	108.8
C16—C24—H24A	108.3	C1—N1—C3	118.7 (2)
C25—C24—H24A	108.3	C1—N1—Cr1	109.93 (19)
C26—C24—H24A	108.3	C3—N1—Cr1	131.32 (19)
C24—C25—H25A	109.5	C2—N2—C15	120.3 (2)
C24—C25—H25B	109.5	C2—N2—Cr1	110.54 (19)
H25A—C25—H25B	109.5	C15—N2—Cr1	128.58 (18)
C24—C25—H25C	109.5	C27—N3—C29	116.0 (2)
H25A—C25—H25C	109.5	C27—N3—Cr2	111.96 (19)
H25B—C25—H25C	109.5	C29—N3—Cr2	131.64 (18)
C24—C26—H26A	109.5	C28—N4—C41	115.3 (2)
C24—C26—H26B	109.5	C28—N4—Cr2	111.59 (18)
H26A—C26—H26B	109.5	C41—N4—Cr2	132.33 (19)
C24—C26—H26C	109.5	C53—O1—C56	109.0 (2)
H26A—C26—H26C	109.5	C53—O1—Cr2	127.10 (16)
H26B—C26—H26C	109.5	C56—O1—Cr2	123.88 (16)
N3—C27—C28	116.5 (3)	Cr2—Cl2—Cr1	81.29 (3)
N3—C27—H27A	121.8	Cr2—Cl3—Cr1	81.07 (2)
C28—C27—H27A	121.8	Cr2—Cl4—Cr1	80.30 (3)
N4—C28—C27	117.2 (2)	N2—Cr1—N1	81.95 (10)
N4—C28—H28A	121.4	N2—Cr1—Cl1	92.03 (7)
C27—C28—H28A	121.4	N1—Cr1—Cl1	90.83 (7)
C30—C29—C34	120.9 (3)	N2—Cr1—Cl3	92.11 (7)
C30—C29—N3	119.0 (3)	N1—Cr1—Cl3	97.04 (7)
C34—C29—N3	120.0 (3)	Cl1—Cr1—Cl3	171.55 (3)
C29—C30—C31	118.3 (3)	N2—Cr1—Cl2	172.58 (7)
C29—C30—C38	123.2 (3)	N1—Cr1—Cl2	98.48 (8)
C31—C30—C38	118.5 (3)	Cl1—Cr1—Cl2	95.36 (3)
C32—C31—C30	121.6 (3)	Cl3—Cr1—Cl2	80.48 (3)
C32—C31—H31A	119.2	N2—Cr1—Cl4	99.39 (7)
C30—C31—H31A	119.2	N1—Cr1—Cl4	178.63 (8)
C31—C32—C33	119.6 (3)	Cl1—Cr1—Cl4	88.84 (3)
C31—C32—H32A	120.2	Cl3—Cr1—Cl4	83.22 (3)
C33—C32—H32A	120.2	Cl2—Cr1—Cl4	80.24 (3)
C32—C33—C34	121.4 (3)	N3—Cr2—N4	81.34 (10)
C32—C33—H33A	119.3	N3—Cr2—O1	93.49 (9)
C34—C33—H33A	119.3	N4—Cr2—O1	93.29 (8)
C33—C34—C29	118.2 (3)	N3—Cr2—Cl2	96.39 (7)
C33—C34—C35	119.6 (3)	N4—Cr2—Cl2	95.80 (7)
C29—C34—C35	122.1 (3)	O1—Cr2—Cl2	167.46 (6)
C34—C35—C37	112.6 (3)	N3—Cr2—Cl3	95.48 (7)
C34—C35—C36	109.9 (3)	N4—Cr2—Cl3	176.21 (8)
C37—C35—C36	110.7 (3)	O1—Cr2—Cl3	88.96 (6)
C34—C35—H35A	107.8	Cl2—Cr2—Cl3	82.45 (3)
C37—C35—H35A	107.8	N3—Cr2—Cl4	178.65 (7)
C36—C35—H35A	107.8	N4—Cr2—Cl4	97.66 (7)
C35—C36—H36A	109.5	O1—Cr2—Cl4	87.47 (6)
C35—C36—H36B	109.5	Cl2—Cr2—Cl4	82.78 (3)
H36A—C36—H36B	109.5	Cl3—Cr2—Cl4	85.48 (3)
C35—C36—H36C	109.5	Cl7—C58—Cl8	112.1 (3)
H36A—C36—H36C	109.5	Cl7—C58—H58A	109.2
H36B—C36—H36C	109.5	Cl8—C58—H58A	109.2
C35—C37—H37A	109.5	Cl7—C58—H58B	109.2
C35—C37—H37B	109.5	Cl8—C58—H58B	109.2
H37A—C37—H37B	109.5	H58A—C58—H58B	107.9
C35—C37—H37C	109.5	Cl10—C59—Cl9	110.9 (3)
H37A—C37—H37C	109.5	Cl10—C59—H59A	109.5
H37B—C37—H37C	109.5	Cl9—C59—H59A	109.5
C40—C38—C30	112.0 (3)	Cl10—C59—H59B	109.5
C40—C38—C39	110.4 (3)	Cl9—C59—H59B	109.5
C30—C38—C39	110.9 (3)	H59A—C59—H59B	108.0
C40—C38—H38A	107.8	Cl12—C60—Cl11	112.0 (3)
C30—C38—H38A	107.8	Cl12—C60—H60A	109.2
C39—C38—H38A	107.8	Cl11—C60—H60A	109.2
C38—C39—H39A	109.5	Cl12—C60—H60B	109.2
C38—C39—H39B	109.5	Cl11—C60—H60B	109.2
H39A—C39—H39B	109.5	H60A—C60—H60B	107.9
C38—C39—H39C	109.5	Cl13—C61—Cl14	117.7 (5)
H39A—C39—H39C	109.5	Cl13—C61—H61A	107.9
H39B—C39—H39C	109.5	Cl14—C61—H61A	107.9
C38—C40—H40A	109.5	Cl13—C61—H61B	107.9
C38—C40—H40B	109.5	Cl14—C61—H61B	107.9
H40A—C40—H40B	109.5	H61A—C61—H61B	107.2
C38—C40—H40C	109.5	Cl6A—C57—Cl5	112.5 (5)
H40A—C40—H40C	109.5	Cl6B—C57—Cl5	114.1 (5)
H40B—C40—H40C	109.5	Cl6A—C57—H57A	109.1
C46—C41—C42	121.6 (3)	Cl6B—C57—H57A	90.2
C46—C41—N4	120.6 (3)	Cl5—C57—H57A	109.1
C42—C41—N4	117.8 (3)	Cl6A—C57—H57B	109.1
C43—C42—C41	117.8 (3)	Cl6B—C57—H57B	123.9
C43—C42—C50	118.7 (3)	Cl5—C57—H57B	109.1
C41—C42—C50	123.5 (3)	H57A—C57—H57B	107.8
C44—C43—C42	121.2 (3)	Cl6A—C57—H57C	125.5
C44—C43—H43A	119.4	Cl6B—C57—H57C	108.7
C42—C43—H43A	119.4	Cl5—C57—H57C	108.7
C43—C44—C45	120.3 (3)	H57B—C57—H57C	88.6
C43—C44—H44A	119.8	Cl6A—C57—H57D	91.2
C45—C44—H44A	119.8	Cl6B—C57—H57D	108.7
C44—C45—C46	121.4 (3)	Cl5—C57—H57D	108.7
C44—C45—H45A	119.3	H57A—C57—H57D	125.0
C46—C45—H45A	119.3	H57C—C57—H57D	107.6
